# The ability of Oxygen Reserve Index® to detect hyperoxia in critically ill patients

**DOI:** 10.1186/s13613-022-01012-w

**Published:** 2022-05-16

**Authors:** Hugues de Courson, Thomas Julien-Laferrière, Delphine Georges, Philippe Boyer, Eric Verchère, Matthieu Biais

**Affiliations:** 1 Department of Anesthesiology and Critical Care, Bordeaux University Hospital, Bordeaux, France; 2 Institut National de la Santé et de la Recherche Médicale, UMR 1219, Bordeaux Population Health Research Center, CHU Bordeaux, Université de Bordeaux, Bordeaux, France; 3 Biology of Cardiovascular Diseases, Institut National de la Santé et de la Recherche Médicale, U1034, Pessac, France

**Keywords:** Oxygen, ORI, Arterial oxygen tension, Hyperoxia, Hypoxemia

## Abstract

**Background:**

Hyperoxia is associated with increased morbidity and mortality in the intensive care unit. Classical noninvasive measurements of oxygen saturation with pulse oximeters are unable to detect hyperoxia. The Oxygen Reserve Index (ORI) is a continuous noninvasive parameter provided by a multi-wave pulse oximeter that can detect hyperoxia. Primary objective was to evaluate the diagnostic accuracy of the ORI for detecting arterial oxygen tension (PaO_2_) > 100 mmHg in neurocritical care patients. Secondary objectives were to test the ability of ORI to detect PaO_2_ > 120 mmHg and the ability of pulse oximetry (SpO_2_) to detect PaO_2_ > 100 mmHg and PaO_2_ > 120 mmHg.

**Methods:**

In this single-center study, we collected ORI and arterial blood samples every 6 h for 3 consecutive days. Diagnostic performance was estimated using the area under the receiver operating characteristic curve (AUROC).

**Results:**

There were 696 simultaneous measurements of ORI and PaO_2_ in 62 patients. Considering the repeated measurements, the correlation between ORI and PaO_2_ was *r* = 0.13. The area under the receiver operating characteristic curve (AUROC), obtained to test the ability of ORI to detect PaO_2_ > 100 mmHg, was 0.567 (95% confidence interval = 0.566–0.569) with a sensitivity of 0.233 (95%CI = 0.230–0.235) and a specificity of 0.909 (95%CI = 0.907–0.910). The AUROC value obtained to test the ability of SpO_2_ to detect a PaO_2_ > 100 mmHg was 0.771 (95%CI = 0.770–0.773) with a sensitivity of 0.715 (95%CI = 0.712–0.718) and a specificity of 0.700 (95%CI = 0.697–0.703). The diagnostic performance of ORI and SpO_2_ for detecting PaO_2_ > 120 mmHg was AUROC = 0.584 (95%CI = 0.582–0.586) and 0.764 (95%CI = 0.762–0.766), respectively. The AUROC obtained for SpO_2_ was significantly higher than that for ORI (*p* < 0.01). Diagnostic performance was not affected by sedation, norepinephrine infusion, arterial partial pressure of carbon dioxide, hemoglobin level and perfusion index.

**Conclusion:**

In a specific population of brain-injured patients hospitalized in a neurointensive care unit, our results suggest that the ability of ORI to diagnose hyperoxia is relatively low and that SpO_2_ provides better detection.

**Supplementary Information:**

The online version contains supplementary material available at 10.1186/s13613-022-01012-w.

## Introduction

In the late 2010s, there is growing interest in the medical literature in detecting of hyperoxia that goes undetected by the use of pulse oximeters. It has deleterious effects in numerous settings, including critical care, and has been associated with increased morbidity and mortality [1–6]. In a specific group of brain-injured patients, where oxygenation is an important goal, a study of traumatic brain injury has shown that hyperoxia increases mortality compared to normoxia, but hyperoxia was set at a very high level (PaO_2_ > 300 mmHg or 40 kPa) [7]. More recently, the European Society of Intensive Care Medicine has recommended that in acute brain injury with or without intracranial pressure rise, the optimal PaO_2_ range to meet metabolic needs is 80–120 mmHg (10–16 kPa) [8]. Thus, because of the narrow therapeutic range, monitoring hyperoxia may be important in this patient population. To date, the diagnosis of hyperoxia requires arterial blood sampling to measure partial pressure of oxygen (PaO_2_), which remains invasive and can lead to increased endoluminal contamination with repeated sampling. Arterial blood gas analyses are intermittent and do not allow continuous measurement of PaO_2_.

MASIMO (Irvine, USA) has developed a noninvasive, multi-wave pulse oximeter called Radical-7®. This device incorporates a conventional pulse oximeter in conjunction with two other measurements, the perfusion index (PI) and the Oxygen Reserve Index (ORI). PI is based on the ratio of pulsatile to non-pulsatile blood flow and should reflect peripheral perfusion [9]. The ORI uses the Fick equation, arterial oxygen content, and venous oxygen saturation (SvO_2_). Indeed, arterial oxygen saturation (SaO_2_) increases to a maximum of 100% in hyperoxia, while SvO_2_ continues to increase by 80% at a PaO_2_ of 200 mmHg [10]. Thus, assuming that cardiac output and oxygen consumption are constant, SvO_2_ is directly proportional to PaO_2_, and ORI should detect changes in SvO_2_ when SaO_2_ reaches its maximum.

In a population of healthy volunteers and patients under general anesthesia in the operating room, ORI seems to be related to PaO_2_ and could also predict desaturation before pulse oximetry (SpO_2_) [11–13]_._ In ICU patients, the results are still unclear because several factors (vasomotor tone, use of vasopressor, degree of sedation) may alter the plethysmographic signal. Although ORI cannot replace arterial blood gas analysis, it could avoid hyperoxia thanks to its continuous monitoring and a specific protocol to lower the percentage of inspired oxygen. In the context of our study evaluating ORI in patients with acute brain injury, two thresholds for hyperoxia can be discussed: 100 mmHg as proposed by Massimo for ORI and 120 mmHg according to the recommendations of the European Society of Intensive Care Medicine in brain-injured patients.

The primary objective of the study was to evaluate the diagnostic accuracy of ORI for detecting PaO_2_ > 100 mmHg. Secondary objectives were to evaluate the ability of ORI to detect PaO_2_ > 120 mmHg, the ability of SpO_2_ to detect PaO_2_ > 100 mmHg and PaO_2_ > 120 mmHg and to evaluate the relationship between ORI and PaO_2_ in the ICU.

## Materials and methods

### Study

We performed this observational, single-center study in the neurological ICU of the Bordeaux University Hospital. This study was approved by the Institutional Review Board (CERAR IRB 00010254-2018-119). According to French law, written consent was not required, and patients and/or their next of kin were informed that their anonymized data would be included in the database. No one refused to participate.

### Patients

The inclusion criteria were patients aged 18 years or older, admitted to the medical-surgical neurological ICU, who had arterial access and a pulse oximetry sensor on the patient middle or index finger (RD Rainbow Lite SET™, Masimo, Irvine, CA), connected to a Radical-7 pulse oximeter (Masimo, Irvine, CA), and who had a blood gas measurement every 6 h according to the local protocol (GEM® Premier™ 5000, Instrumentation Laboratory, Bedford, MA, United States). There were no restrictions on ventilation mode or drug infusion.

Data were collected every 6 h for 3 consecutive days. We collected measurements of Radical-7®: SpO_2_, ORI (after visual inspection of the quality and stability of the signal) and PI at the time of blood gas sampling, as well as an arterial blood gas sample.

We also collected: demographic data, reason for ICU admission, Simplified Acute Physiology Score (SAPS II); ventilatory settings (in patients with a simple nasal cannula, FiO_2_ was estimated as follows: oxygen flow (l/min) × 3 + 21) [15]; neurological status data: Richmond Agitation Sedation Scale (RASS) if subjects were sedated or Glasgow Coma Scale (GCS) if not, and hemodynamic data. Finally, we collected information on the use of commonly used drugs that might affect vasomotion (remifentanil, sufentanil, propofol, midazolam, norepinephrine, milrinone, urapidil and nicardipine).

### Statistical analysis

Because of insufficient data on ORI, no sample size calculation was performed. We chose to include every patient who met the inclusion criteria for one year. Based on usual recruitment we assumed that we would include more than 50 patients. Continuous data were reported as mean (standard deviation) or median [interquartile range] depending on the distribution of the data. Qualitative data were expressed as number (percentage). The correlation between PaO_2_ and ORI was crudely estimated first and considering repeated measurements second [16]. Diagnostic performance included the ability of the ORI to detect a PaO_2_ greater than 100 mmHg and greater than 120 mmHg. We also evaluated the ability of SpO_2_ to detect a PaO_2_ greater than 100 mmHg and greater than 120 mmHg. Diagnostic performance was estimated by the area under the receiver operating characteristic curve (AUROC), positive predictive value (PPV), and negative predictive value (NPV). Because of repeated measurements, 95% confidence intervals (CI) around the parameters were estimated using a 1000 individual bootstrap. The influence of different factors such as PaCO_2_, hemoglobin, perfusion index, or norepinephrine dose on the diagnostic performance of ORI was estimated by using the semiparametric approach proposed by Farragi et al. [17]. The data analysis and the statistical plan were written and submitted to the Institutional Review Board (CERAR IRB 00010254-2018-119) before accessing the data. All analyses were performed using R version 4.0.3 (Vienne, Austria).

## Results

### Patients

**Fig. 1:**
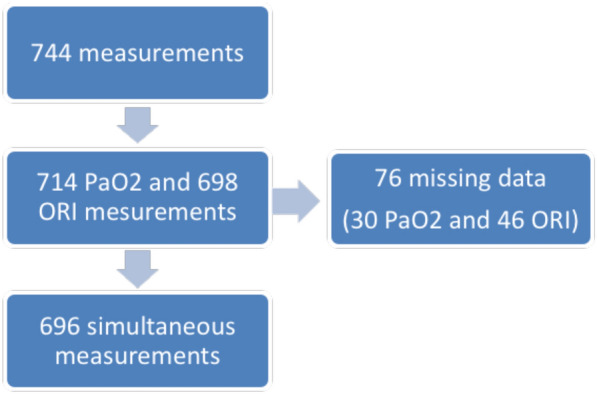
Study flowchart

We included 62 patients between August 2018 and August 2019. The main characteristics are listed in Table 1. Patients had a high SAPS 2 score (mean: 43), were frequently hospitalized after subarachnoid hemorrhage (66%), and were intubated in 53% of cases. Subjects were included in the early days of hospitalization in ICU (median: 3rd). Measurements were mostly performed without sedation. Of the 744 measurements, 714 PaO_2_ (242 of which > 100 mmHg and 82 of which > 120 mmHg) and 698 ORI values (98 of which > 0.0) were recorded with a total of 696 simultaneous measurements (Fig. 1). All ORI values were included, regardless of the PI value. Missing data were not considered in the statistical analysis.

**Table 1:** Patients characteristics (*n* = 62)

Age (years)	57 (13)
Sex	
Female	40 (66)
Body mass index (kg/m^2^)	26 (5)
Reason for admission	
Sub-arachnoid hemorrhage	41 (66)
Hemorrhagic stroke	13 (21)
Ischemic stroke	3 (5)
Other	5 (8)
Preexisting condition	
High blood pressure	30 (48%)
Coronary heart disease	3 (5%)
Active tobacco smoking	24 (39%)
Dyslipidemia	9 (15%)
Obesity (body mass index > 30 kg/m^2^)	12 (19%)
Chronic obstruction pulmonary disease	3 (5%)
Peripheral arterial disease	3/(5%)
SAPS II score	43 (19)
Intubation	
Yes	47 (76)
Delay between admission and inclusion	3 [2–5]

Data are expressed as mean (standard deviation), median [25–75% interquartile range] or count (percentage%) as appropriate

*SAPS* Simplified Acute Physiology Score

### Correlation between ORI and PaO_2_

**Fig. 2:**
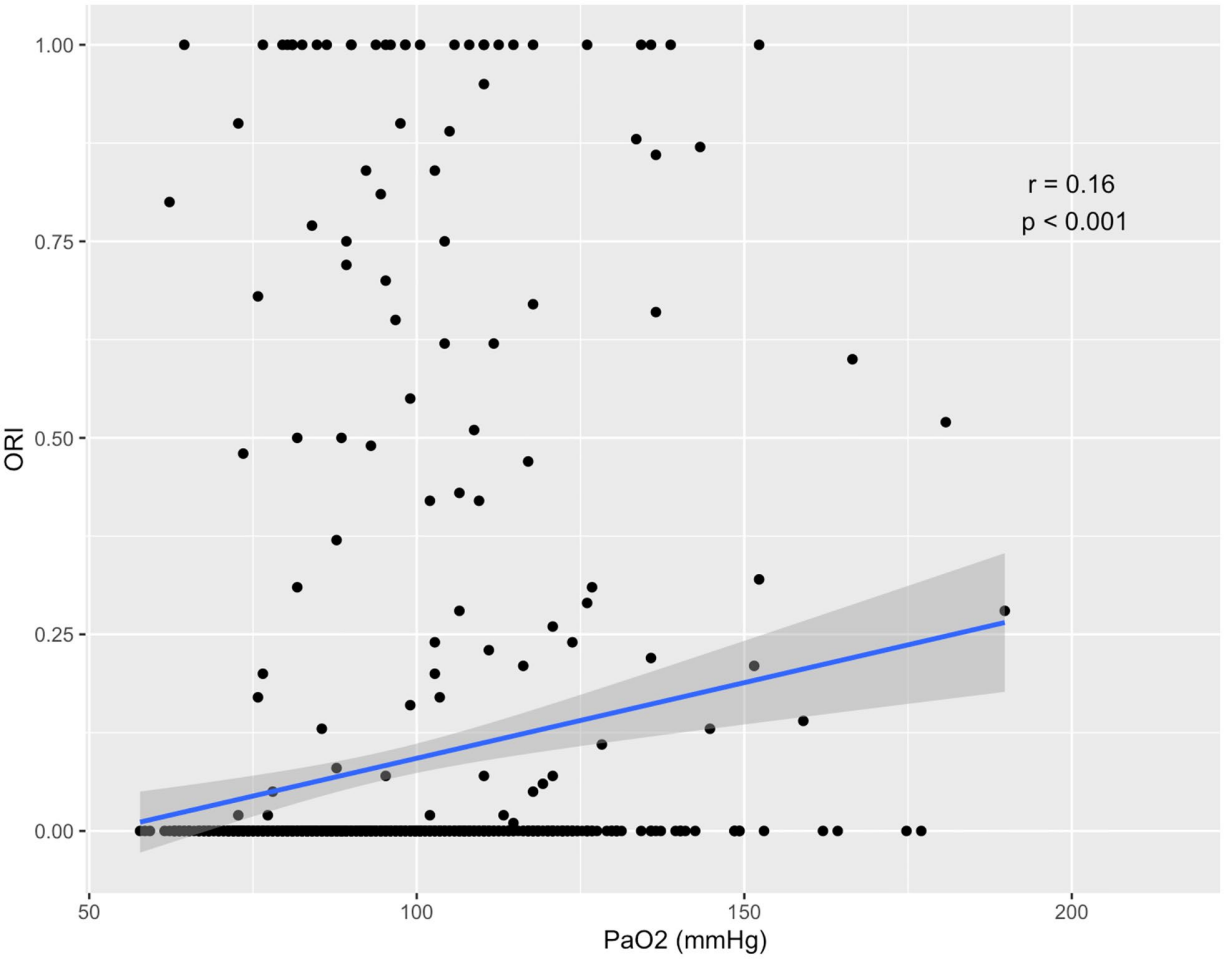
Global correlation between oxygen partial pressure (PaO_2_) and Oxygen Reserve Index

Blood gas analysis and ORI data are shown in Table 2. The conditions during the measurements are listed in Table 3. For all measurements, crude correlation between ORI and PaO_2_ was *r* = 0.16; *p* < 0.001 (Fig. 2); when repeated measurements were taken into account correlation was *r* = 0.13; *p* < 0.001 (Additional file 1: Fig. S1).

**Table 2:** Blood gas analysis and ORI data

	Number of data analyzed	Mean (standard deviation) or number (%)
Pulse oximetry (%)	712	97 (2)
Oxygen Reserve Index (no units)	698	0.08 (0.25)
Number of Oxygen Reserve Index measurements > 0	698	99 (14)
Perfusion index (no units)	682	2.9 (1.8)
pH (no units)	716	7.45 (0.04)
PaO_2_ (mmHg)	714	96 (23)
PaCO_2_ (mmHg)	715	38 (5)
Hemoglobin (g/dl)	699	11.0 (1.7)
Number of PaO_2_ measurements > 100mHg	714	244 (34)

Data are expressed as mean (standard deviation)

*PaO*_*2*_ partial pressure of oxygen

*PaCO*_*2*_ partial pressure of carbon dioxide

**Table 3:** Measurement conditions

	Number (percentage) or median [25–75% interquartile range]
Intubation	
Yes	391 (53)
No	341 (47)
Ventilation mode (If intubated)	
Controlled	181 (46)
Spontaneous	210 (54)
Ventilation mode (If not intubated)	
Non-invasive ventilation	8 (2)
High-flow nasal cannula oxygen	34 (10)
Nasal cannula	299 (88)
Mean arterial pressure (mmHg)	93 (22)
Heart rate (BPM)	79 (19)
Pulse pressure (mmHg)	78 (18)
Norepinephrine infusion	
Yes	218 (32)
Dose (μg/kg/min)	0.39 (0.33)
Milrinone	
Yes	67 (10)
Dose (μg/kg/min)	1.9 [0.8–2.0]
Urapidil	
Yes	84 (12)
Dose (mg/h)	50 [20–60]
Nicardipine	
Yes	28 (4)
Dose (mg/h)	8 [6–9]
Sedation	
Yes	101 (15)
No	585 (85)
RASS score (if sedation)	
− 5	83 (86)
− 4	13 (13)
− 3 to 2	1 (1)
Glasgow score (If no sedation)	
3–7	32 (8)
8–11	103 (26)
11–15	259 (66)
Propofol	
Yes	59 (9)
Dose (mg/h)	200 [120–240]
Sufentanil	
Yes	117 (17)
Dose (μg/h)	40 [20–60]
Midazolam	
Yes	59 (9)
Dose (mg/h)	10 [5–20]

### The ability of ORI and SpO2 to predict hyperoxia

**Fig. 3:**
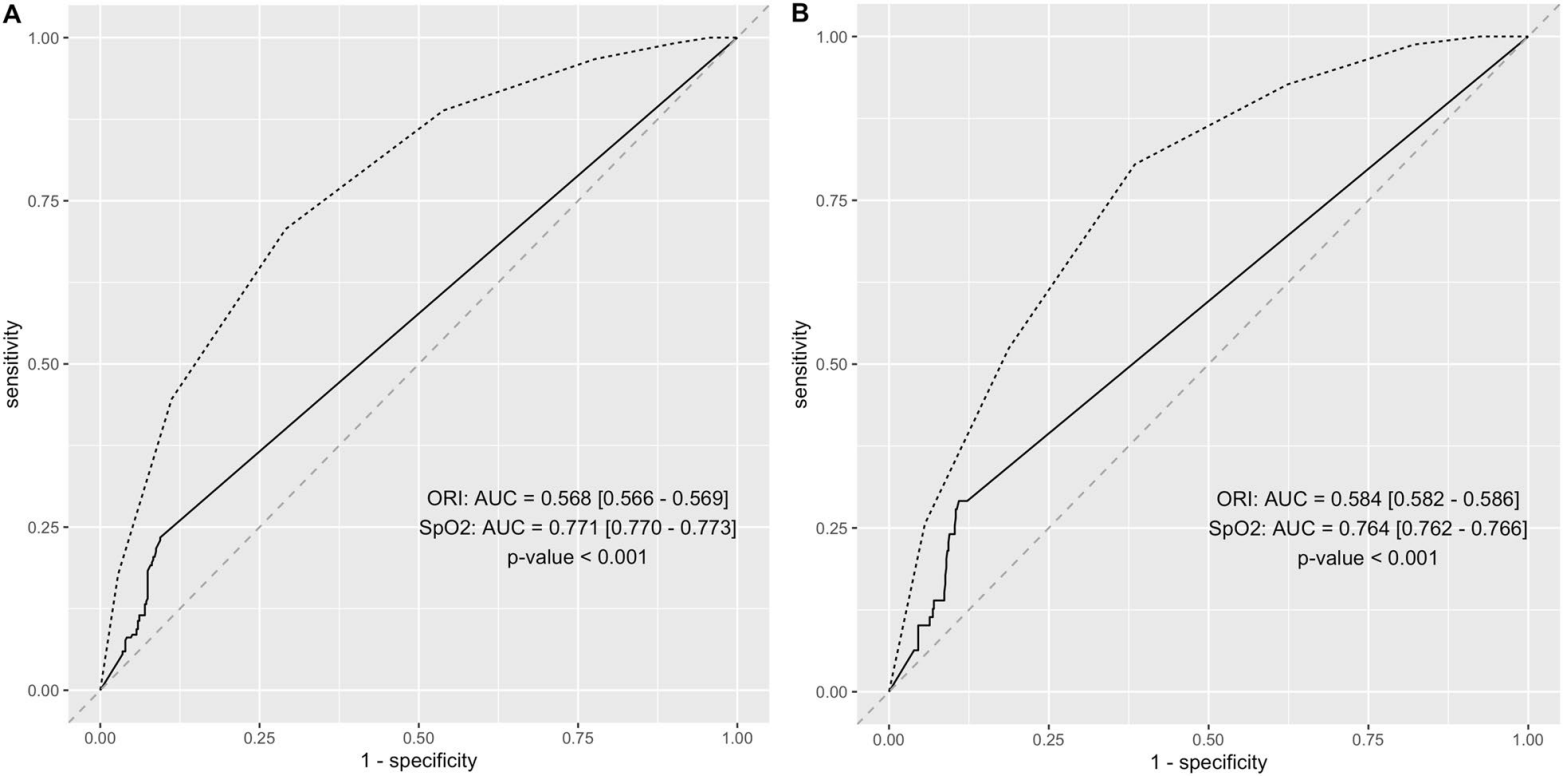
Receiving operating characteristics (ROC) curves. **A** Abilites of Oxygen Reserve Index (ORI) and pulse oxyemtry (SpO_2_) to predict oxygen partial pressure (PaO_2_) > 100 mmHg. **B** Abilites of Oxygen Reserve Index (ORI) and pulse oxyemtry (SpO_2_) to predict oxygen partial pressure (PaO_2_) > 120 mmHg

The ability of ORI to predict PaO_2_ > 100 mmHg was low; AUROC = 0.567 [95% CI = 0.566–0.569] (Fig. 3). The abilities of ORI to predict PaO_2_ > 120 mmHg and the ability of SpO_2_ to predict hyperoxia, defined by PaO_2_ > 100 mmHg and > 120 mmHg are shown in Table 4. AUROC, which was generated for SpO_2_, was significantly different from AUROC generated for ORI in order to detect hyperoxia (Fig. 3). An SpO_2_ value less than or equal to 94% allows a PaO_2_ > 100 mmHg to be excluded with a sensitivity of 100%. An SpO_2_ value less than or equal to 95% allows to exclude with a sensitivity of 100% a PaO_2_ > 120 mmHg. Diagnostic performance did not appear to be affected by sedation, norepinephrine infusion, PaCO_2_ value, hemoglobin value and perfusion index (Additional file 2: Fig. S2, Additional file 3: Fig. S3).

**Table 4:** Diagnostic performance of ORI and SpO_2_ to detect PaO_2_ > 100 mmHg and PaO_2_ > 120 mmHg

Parameter	Hyperoxia threshold	AUROC [CI95%]	Youden index	Best threshold	Sensitivity [CI95%]	Specificity [CI95%]	PPV [CI95%]	NPV [CI95%]
ORI	100 mmHg	0.567 [0.566–0.569]	0.140	0.005	0.233 [0.230–0.235]	0.907 [0.907–0.910]	0.567 [0.563–0.571]	0.696 [0.694–0.698]
SpO_2_	100 mmHg	0.771 [0.770–0.773]	0.416	97.5	0.715 [0.712–0.718]	0.700 [0.697–0.703]	0.555 [0.552–0.558]	0.826 [0.824–0.828]
ORI	> 120 mmHg	0.584 [0.582–0.586]	0.182	0.065	0.289 [0.285–0.294]	0.892 [0.891–0.893]	0.257 [0.254–0.260]	0.906 [0.905–0.907]
SpO2	> 120 mmHg	0.764 [0.762–0.766]	0.420	97.5	0.799 [0.795–0.803]	0.623 [0.620–0.626]	0.218 [0.216–0.220]	0.959 [0.958–0.960]

*AUROC* area under the receiver operating characteristic curve, *NPV* negative predictive value, *ORI* Oxygen Reserve Index, *PPV* positive predictive value, *SpO*_*2*_: pulse oximetry

## Discussion

Our results, based on a large number of simultaneous measurements of PaO_2_ and ORI performed in a neuro-ICU population, show that: (i) the ability of ORI to detect hyperoxia (defined by PaO_2_ > 100 mmHg or PaO_2_ > 120 mmHg) was low; (ii) the diagnostic performance of SpO_2_ to detect hyperoxia was better than that of ORI, and that (iii) ORI and PaO_2_ were poorly correlated. Diagnostic performance did not appear to be affected by patient conditions such as perfusion index, use of vasoactive drugs such as norepinephrine, or metabolic parameters (PCO_2_ and hemoglobin). We chose to investigate two PaO_2_ thresholds. The definition of hyperoxia is not uniform and it seemed interesting to us to study the threshold proposed by Masimo for the capacities of the ORI (100 mmHg) and the threshold used in our population of brain-injured patients (120 mmHg).

Several studies suggest that hyperoxia may increase both morbidity and mortality. In a large multicenter cohort study of patients resuscitated from cardiac arrest, excessive oxygen tension was associated with an increased risk of in-hospital death [18]. A meta-analysis that included 25 randomized control trials involving more than 16,000 patients with sepsis, critical illness, stroke, trauma, myocardial infarction, or cardiac arrest, as well as patients undergoing emergency surgery, showed that liberal oxygen therapy increased mortality without improving other outcomes important to the patient [19]. Other work has found that short-term hyperoxia after intubation in the emergency department was associated with increased mortality in the intensive care unit [20]. Randomized controlled trials have shown that lower oxygenation targets (PaO_2_ < 100 mmHg (< 13 kPa) and SpO_2_ < 97%) are associated with higher survival rates and shorter duration of mechanical ventilation in ICU patients [21, 22]. However, the data are not entirely conclusive, as some studies have reached different conclusions. Two recent randomized control trials failed to find a benefit of normoxia compared with moderate hyperoxia [23, 24].

Under study conditions, we found no association between ORI and PaO_2_ levels. Although we acknowledge that the ORI is not marketed as a noninvasive PaO_2_ monitor, our results are not consistent with previous studies. This may be explained by several factors. Several positive studies have focused on the ability of the ORI to predict desaturations in the operating room, in pediatrics, in emergencies, in obese patients, or in thoracic surgery [14, 25–29]. These results are very interesting, but address a completely different question than ours (desaturation versus prediction of hyperoxia). Other works found a good correlation between ORI and PaO_2_. These studies included healthy volunteers or surgical patients under general anesthesia [10–13]. In a study of 20 healthy volunteers, Vos et al. found a strong and positive correlation between ORI and PaO_2_ (*R* = 0.8) [13]. In studies conducted in the operating room, a correlation between ORI and PaO_2_ was found, ranging from *r*^2^ = 0.54 to *r*^2^ = 0.71 (12,13). In another study, ORI was found to be able to detect PaO_2_ > 100 mmHg (threshold = 0.24) [12]. Compared to patients under general anesthesia in the operating room, patients in the ICU differ in many ways. General inflammation can cause peripheral edema, which can alter the plethysmographic signal. ICU patients are more likely to receive vasoactive medications that may alter vasomotor tone, but they do not appear to alter the diagnostic performance of ORI in our study. Anesthesia patients are also all perfectly quiet, which is not the case in the ICU; this element could also explain a signal alteration in the ICU. All these elements could partially explain our results.

ORI has been much less studied in the ICU. To our knowledge, the only study that investigated ORI in the ICU found a significant reduction in hyperoxia when using an ORI-based protocol [30]. In this monocentric study, patients with ORI monitoring were more likely to be ventilated with a FiO2 of 0.21, which probably secured the main outcome. The results of our study suggest that ORI does not seem to be suitable for detecting hyperoxia situations in the ICU. Moreover, there seem to be very large individual variations (Fig. 2).

Our study has several limitations. First, we measured a PaO_2_ greater than 100 mmHg in 34% of cases, which is less than in other studies [4, 20, 31]. However, the sensitivity and specificity of a test are not affected by the prevalence of the disease. We may already have restrictive oxygen targets in our ICU because nurses are allowed to lower the inspiratory O_2_ fraction when SpO_2_ is higher than physician orders, and repeated arterial blood gas measurements have been used to lower FiO_2_, which is done routinely. However, lowering FiO_2_ based on ORI was not allowed. In particular, we found a single PaO_2_ measurement higher than 200 mmHg, which precluded analysis of ORI in the presence of high hyperoxia (PaO_2_ > 200 mmHg). Considering the medical literature, it seems unethical to voluntarily increase PaO_2_ in ICU patients to study the correlation. Second, all ORI values were considered, regardless of the PI value. This can be considered a limitation since a minimum PI is required to obtain adequate ORi measurements. Third, we have 46 missing data, which is low given the number of measurements we performed, but they may have been emergencies in unstable patients in whom our team could have increased FiO_2_. Finally, our results focused on a very specific population of brain-injured patients, mostly after subarachnoid hemorrhage, for whom PaO_2_ values are challenging in both respects. The results remain to be confirmed in other populations.

## Conclusion

Under the conditions of the study, our results suggest that ORI is unable to detect hyperoxia and that ORI and PaO_2_ are poorly correlated in neurocritical care patients.

## Supplementary Information

**Additional file 1: Fig. S1.** Repeated-measurements correlation between oxygen partial pressure (PaO_2_) and Oxygen Reserve Index.

**Additional file 2: Fig. S2.** Area Under the Receiving operating characteristics (AUROC) curves according to norepinephrine dosage and dioxide carbon arterial partial pressure (PaCO_2_) value. Hyperoxemia defined by a PaO_2_ > 100 mmHg. Hyperoxemia defined by a PaO_2_ > 120 mmHg

**Additional file 3: Fig. S3. **Area Under the Receiving operating characteristics (AUROC) curves according to hemoglobin et perfusion index. A Hyperoxemia defined by a PaO_2_ > 100 mmHg; B Hyperoxemia defined by a PaO_2_ > 120 mmHg.

## Data Availability

The datasets used and/or analyzed during the current study are available from the corresponding author on reasonable request.

## Abbreviations

AUROC: Area under the receiver operating characteristic curve
CI: Confidence interval
FiO_2_: Fraction of inspired oxygen
ICU: Intensive Care Unit
kPa: Kilopascals
mmHg: Millimeters of mercury
NPV: Negative predictive value
ORI: Oxygen Reserve Index
PaCO_2_: Carbon dioxide arterial partial pressure
PaO_2_: Arterial oxygen tension
PI: Perfusion index
PPV: Positive predictive value
SaO_2_: Arterial oxygen saturation
SAPS II: Simplified Acute Physiology Score
SpO_2_: Pulse oximetry
SvO_2_: Venous oxygen saturation
