# Supplementary figures and images for: The ability of Oxygen Reserve Index® to detect hyperoxia in critically ill patients
Source: Ann Intensive Care. 2022 May 16;12:40. doi: 10.1186/s13613-022-01012-w (PMC9110610; doi:10.1186/s13613-022-01012-w)

# Supplementary File 1

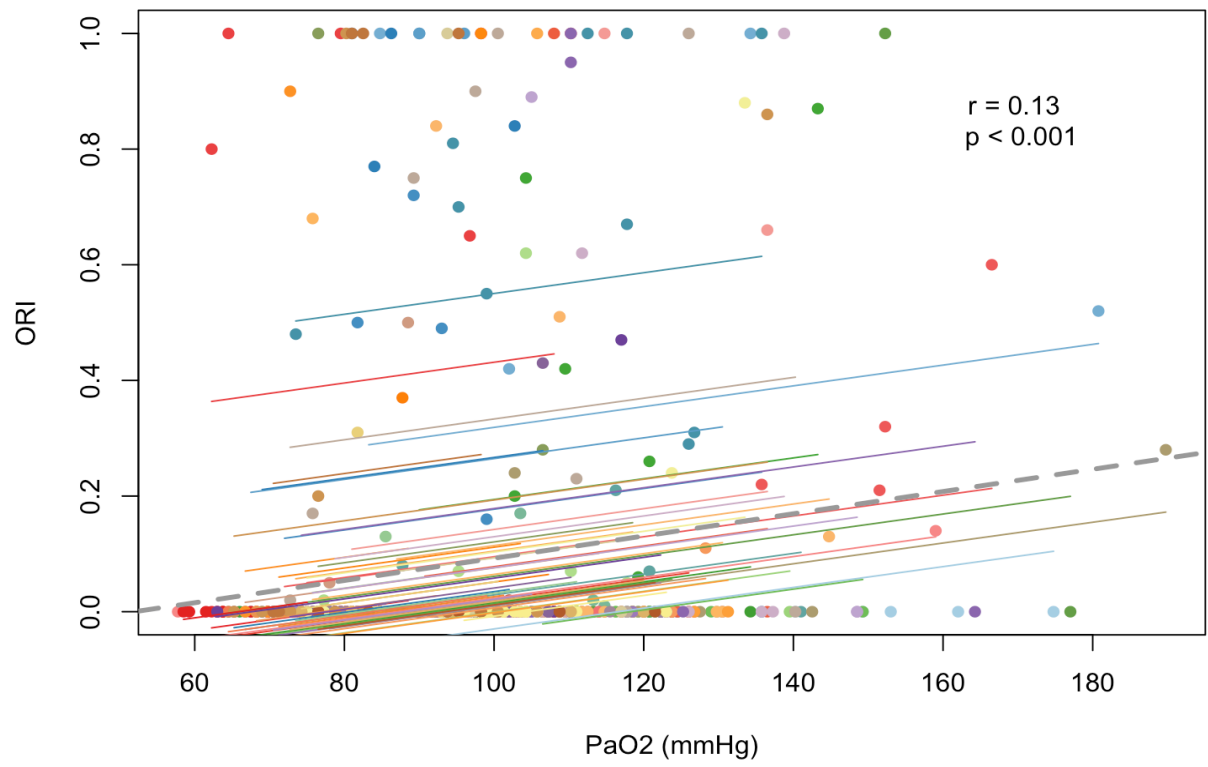

Supplement: Supplementary file 1 — Additional file 1: Fig. S1. Repeated-measurements correlation between oxygen partial pressure (PaO2) and Oxygen Reserve Index. [file 13613_2022_1012_MOESM1_ESM.pdf]

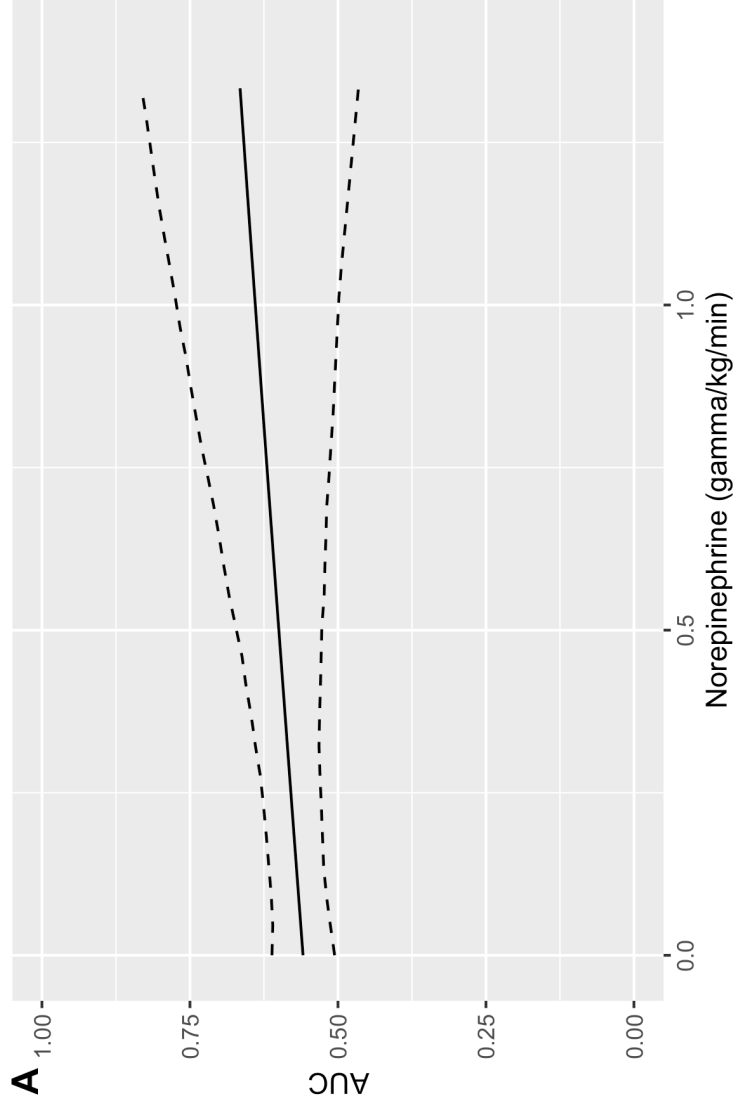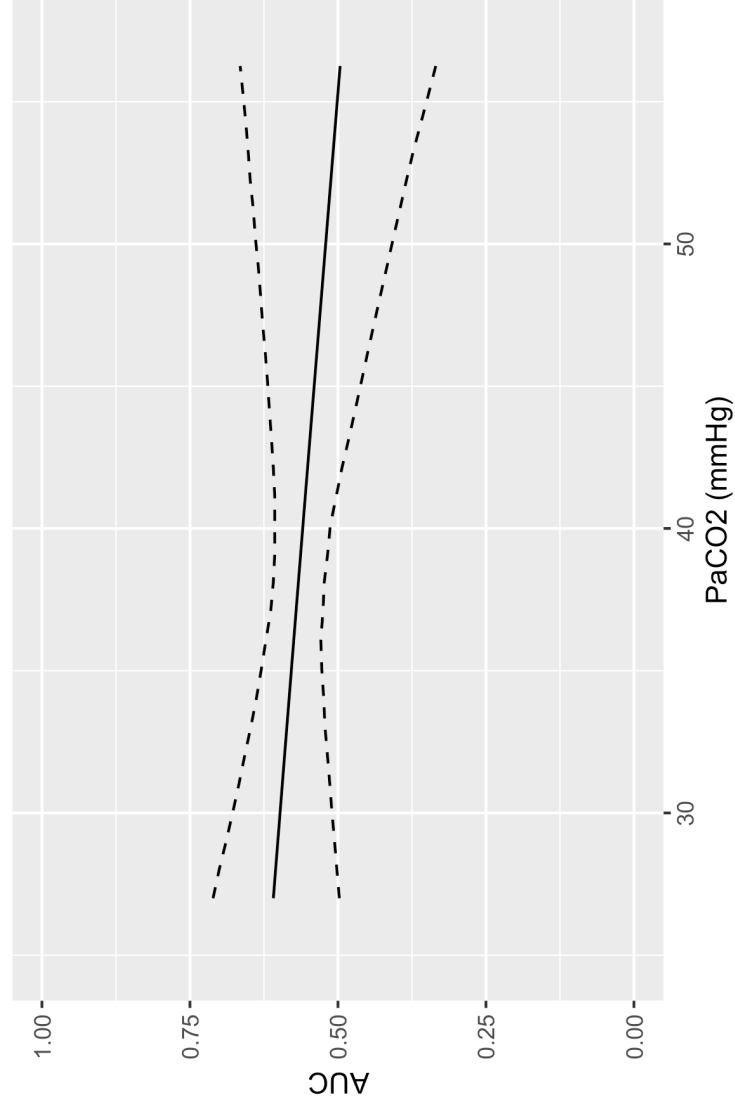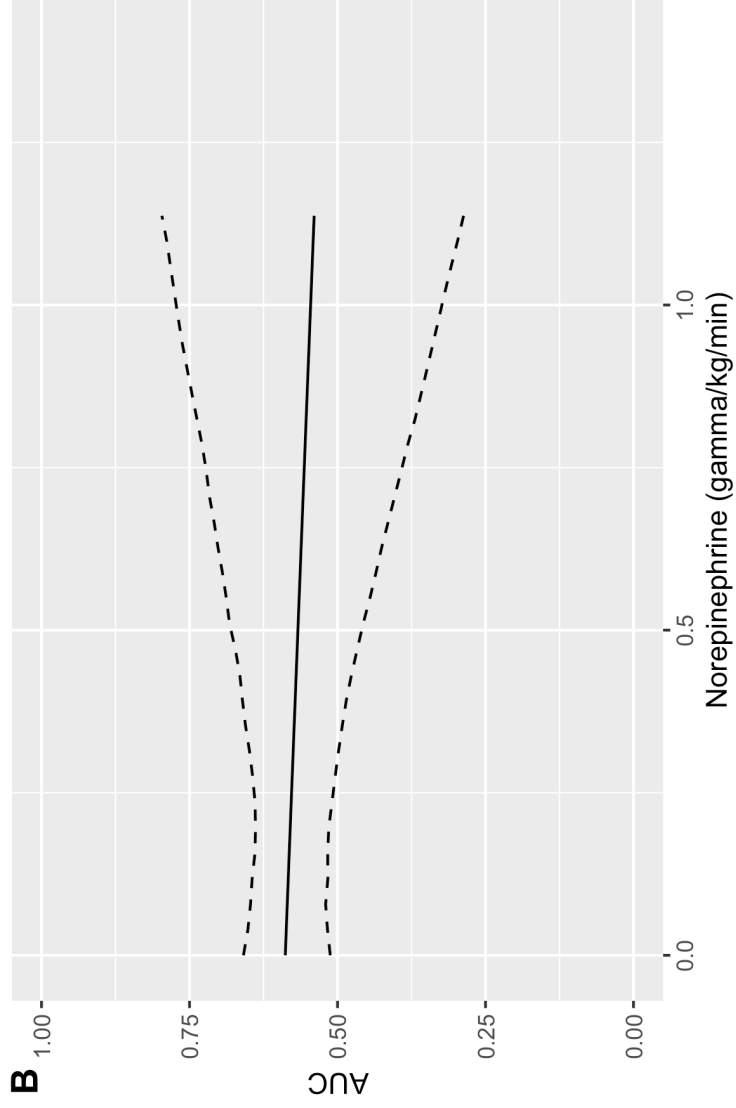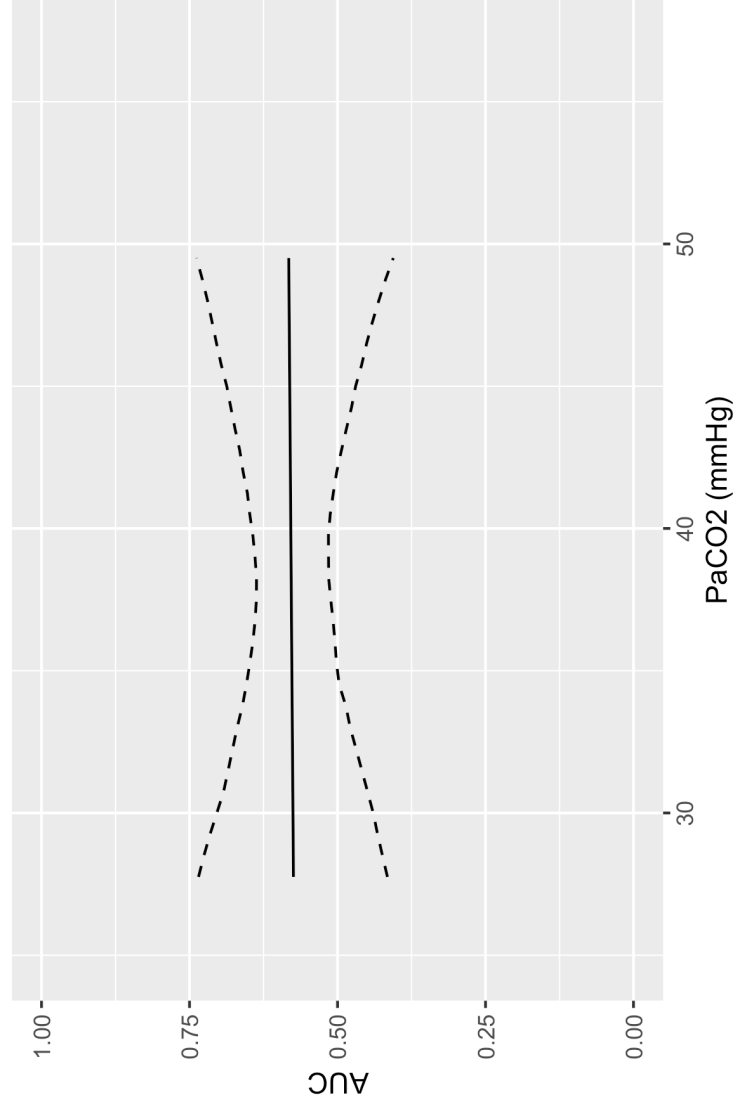

Supplement: Supplementary file 2 — Additional file 2: Fig. S2. Area Under the Receiving operating characteristics (AUROC) curves according to norepinephrine dosage and dioxide carbon arterial partial pressure (PaCO2) value. Hyperoxemia defined by a PaO2 > 100 mmHg. Hyperoxemia defined by a PaO2 > 120 mmHg [file 13613_2022_1012_MOESM2_ESM.pdf]

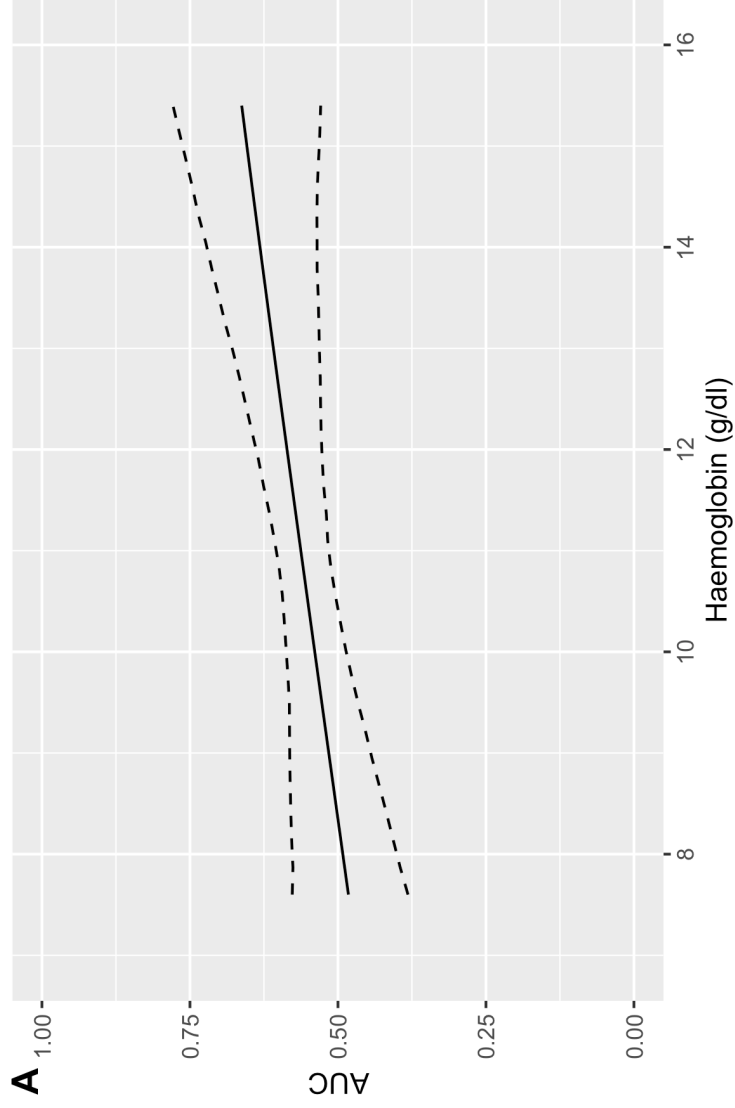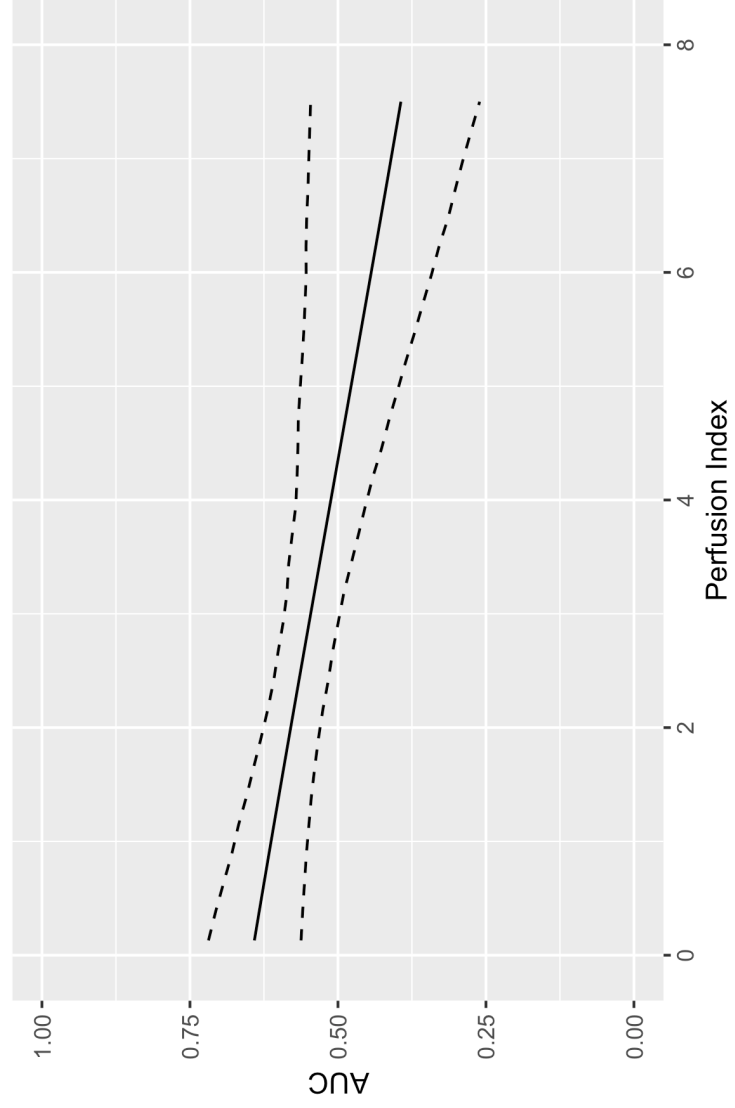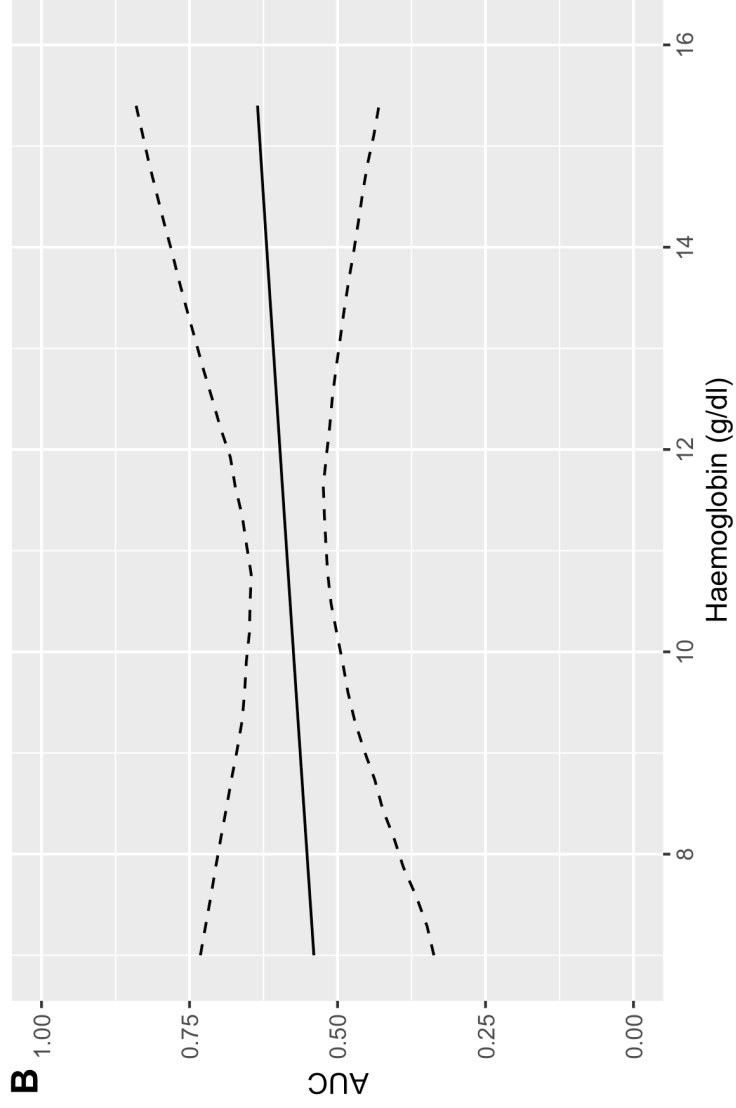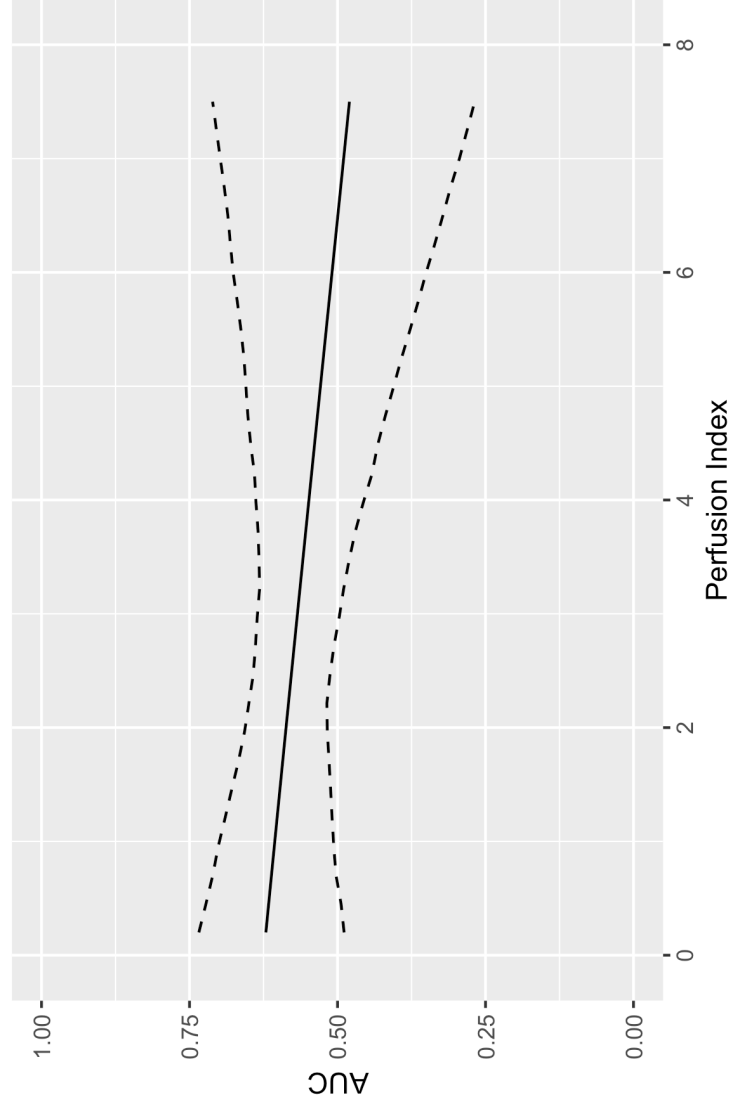

Supplement: Supplementary file 3 — Additional file 3: Fig. S3. Area Under the Receiving operating characteristics (AUROC) curves according to hemoglobin et perfusion index. A Hyperoxemia defined by a PaO2 > 100 mmHg; B Hyperoxemia defined by a PaO2 > 120 mmHg. [file 13613_2022_1012_MOESM3_ESM.pdf]
